# Enrichment of in vivo transcription data from dietary intervention studies with in vitro data provides improved insight into gene regulation mechanisms in the intestinal mucosa

**DOI:** 10.1186/s12263-017-0559-1

**Published:** 2017-04-13

**Authors:** Marcel Hulst, Alfons Jansman, Ilonka Wijers, Arjan Hoekman, Stéphanie Vastenhouw, Marinus van Krimpen, Mari Smits, Dirkjan Schokker

**Affiliations:** 1 Animal Breeding and Genomics Centre, Wageningen University and Research, Wageningen, The Netherlands; 2 Wageningen Livestock Research, Wageningen University and Research, Wageningen, The Netherlands; 3 Wageningen Bioveterinary Research, Wageningen University and Research, Lelystad, The Netherlands

**Keywords:** Zinc, Rye, Amoxicillin, Pigs, Broilers, Intestinal mucosa, Gene expression

## Abstract

**Background:**

Gene expression profiles of intestinal mucosa of chickens and pigs fed over long-term periods (days/weeks) with a diet rich in rye and a diet supplemented with zinc, respectively, or of chickens after a one-day amoxicillin treatment of chickens, were recorded recently. Such dietary interventions are frequently used to modulate animal performance or therapeutically for monogastric livestock. In this study, changes in gene expression induced by these three interventions in cultured “Intestinal Porcine Epithelial Cells” (IPEC-J2) recorded after a short-term period of 2 and 6 hours, were compared to the in vivo gene expression profiles in order to evaluate the capability of this in vitro bioassay in predicting in vivo responses.

**Methods:**

Lists of response genes were analysed with bioinformatics programs to identify common biological pathways induced in vivo as well as in vitro. Furthermore, overlapping genes and pathways were evaluated for possible involvement in the biological processes induced in vivo by datamining and consulting literature.

**Results:**

For all three interventions, only a limited number of identical genes and a few common biological processes/pathways were found to be affected by the respective interventions. However, several enterocyte-specific regulatory and secreted effector proteins that responded in vitro could be related to processes regulated in vivo, i.e. processes related to mineral absorption, (epithelial) cell adherence and tight junction formation for zinc, microtubule and cytoskeleton integrity for amoxicillin, and cell-cycle progression and mucus production for rye.

**Conclusions:**

Short-term gene expression responses to dietary interventions as measured in the in vitro bioassay have a low predictability for long-term responses as measured in the intestinal mucosa in vivo. The short-term responses of a set regulatory and effector genes, as measured in this bioassay, however, provided additional insight into how specific processes in piglets and broilers may be modulated by “early” signalling molecules produced by enterocytes. The relevance of this set of regulatory/effector genes and cognate biological processes for zinc deficiency and supplementation, gluten allergy (rye), and amoxicillin administration in humans is discussed.

**Electronic supplementary material:**

The online version of this article (doi:10.1186/s12263-017-0559-1) contains supplementary material, which is available to authorized users.

## Background

The use of animals to study the beneficial effects of dietary interventions for livestock and humans requires large numbers of test animals. Such experiments are not only expensive but also controversial from an ‘animal welfare’ point of view. Development of in vitro tests that use no test animals, or in situ models that require only a few, could be useful tools to evaluate the prophylactic or therapeutic potentials of ‘novel/alternative’ feed and food additives, ingredients or dietary interventions in a cost-effective manner. Such alternative may complement or replace expensive additives and additives for which ‘restricted’ use is prescribed by authorities, e.g. for antibiotics used to reduce the impact of diarrhoea causing bacteria in the digestive tract of infant animals and humans. Of special interest are novel additives and dietary interventions, which may promote the maturation and competence of the intestinal immune system of the infant. Animals with a well-developed and competent intestinal immune system may eliminate diarrhoea causing pathogens more rapidly, resulting in a faster restoration of the absorptive function of the intestinal epithelial layer.

For in vitro testing of additives and ingredients, we developed an in vitro bioassay based on intestinal porcine epithelial cells (IPEC-J2) [5, 30, 63]. IPEC-J2 cells are derived from mid-jejunum tissue of piglets which are unique, as they are not derived from tumours and thus are not transformed. They closely resemble the digestive and absorptive functions of enterocytes in in vivo conditions which makes them suitable for studying primary interactions of ingredients/additives with enterocytes. Furthermore, IPEC-J2 cells can form a single-cell layer that resembles the architecture of the epithelial layer in vivo, making them also useful for studies regarding the integrity and barrier function of this layer [8, 9]. Finally, IPEC-J2 cells are capable of mounting an immune response after exposure to foreign antigens and pathogens [5, 14, 30], i.e. they are able to express an array of cytokines (IL8, IL1A, IL6, IL7, IL18, TNF and CSF) and several acute-phase response proteins. Moreover, cytokine expression by IPEC-J2 cells in response to enteric pathogens largely reflected that of human, colon-derived, intestinal cell lines (HT29, T84, Caco-2, and SW620) [18]. For the latter reason, IPEC-J2 cells are frequently used to examine primary interactions of enteric pathogens with the epithelial layer. In this study, we used IPEC-J2 cells to mimic the response of mucosal tissues after exposure to ingredients/additives used in animal diets to improve health and performance. However, a bioassay to test the effect of dietary constituents based on in vitro cultured cells has its limitations. In vitro bioassays usually measure an immediate early response. In the IPEC-J2 bioassay, the ideal period for measuring primary responses is not more than 8 h. In vivo, however, long-term adaptive responses, ranging from a few days to several weeks, are most frequently studied in the case of determining the effect of dietary interventions in animals (on local and/or systemic physiologic parameters as well as on zootechnical parameters). An obvious difference between in vitro and in vivo models are the lack of cellular complexity and intercellular signalling in in vitro models based on a single type of cells, such as IPEC-J2. In vivo, there is a variety of interacting cells and cross-talking microbiota, as well as a dynamic (in)flux of different (immune) cells and a blood flow, all of them are absent in the IPEC-J2 system. In addition, a protecting mucus layer on top of the epithelial cell layer is present in vivo, but is not formed in IPEC-J2 cell cultures. On the other hand, enterocytes are the first to respond to changes in the luminal environment and rapidly communicate these changes to the underlying cells in the lamina propia and to the periphery by secreting effector molecules (e.g. cytokines, hormones, free radicals). Therefore, we regard the IPEC-J2 cell line still a valuable tool for screening the primary (early, immediate early) effect of novel and/or alternative feed additives/ingredients.

The objective of the present study is to investigate to what extent gene expression data of dietary interventions generated in an IPEC-J2 in vitro model overlap with in vivo data and whether the immediate early responses, as measured in vitro, provide additional insight into the mechanism and dynamics of ‘dietary modulation’ in vivo. To this end, we measured gene expression responses of IPEC-J2 cells upon exposure with three different dietary interventions commonly used in livestock [31]. In a further step, we compared the results with mucosal gene expression responses, as measured in animals exposed to the same interventions via the diet. As interventions, we used zinc oxide, rye and the antibiotic amoxicillin, three interventions also used and important for human (intestinal) health issues, i.e. for zinc deficiency and supplementation [37], gluten allergy (rye) [64], and antibiotic treatment of bacterial infections.

It was reported that a high dose of zinc could influence the composition and activity of the intestinal microbiota as well as the local immunity in the intestine [6] and reduces the incidence of diarrhoea in weaned piglets [41] and in infants [19]. Dietary inclusion of rye with soluble viscous carbohydrates results in a higher viscosity of the intestinal digesta and may impair nutrient digestibility [4] and was found to stimulate the local influx of immune cells in the intestines of chickens [13, 54]. Furthermore, amoxicillin was found to perturb the ‘normal’ colonisation of the gut microbiota. It was hypothesised that this change in microbiota composition leads to altered immune development. For the in vivo studies with amoxicillin and rye, the results have already been reported and are available in the public domain [50, 57]. For zinc, a manuscript is in preparation [32].

Corresponding in vitro and in vivo gene expression datasets were compared to extract similar response genes, and lists of response genes from all datasets were analysed using bioinformatics programmes to identify similar or closely related pathways enriched in vivo as well as in vitro. In addition, differentially expressed genes (DEGs) in the IPEC-J2 enterocytes, which code for secreted effector proteins (i.e. cytokines/chemokines and activators or inhibitors of transcription cascades), or for enzymes that metabolise chemical effector molecules (e.g. chemical immune modulators), were extracted and reviewed for a possible regulatory role in the biological processes identified in in vivo studies. The relevancy of the overlap in genes/processes observed between the in vitro and in vivo datasets and the role of putative effectors were reviewed by extensive data mining and consulting literature related to the tested interventions. In addition, the relevance and possible applications that emerged from our findings for human (intestinal) health issues are discussed.

## Methods

### Animal intervention studies

A brief description of the setup of the three animal intervention trials is provided in Additional file 1. In Table 1, an overview of noteworthy parameters applied in these animal trials and of the comparison of gene expression profiles from which lists of differentially expressed genes (DEGs) were retrieved is given. Details about the composition of the diets supplemented with dietary interventions, the control diets, sampling of biological material for analysis, isolation of total RNA from these tissues, and microarray analysis and extraction of DEGs were described in reports available online or in the articles published about these trails [32, 50, 57]. The three animal trials were performed according to the national guidelines for the care and use of laboratory animals after approval of the animal welfare committee of Wageningen University under code 2013095.b for zinc, 2013090.b for rye and 2013035.b for amoxicillin.

**Table 1:** Number of DEGs in all in vitro and in vivo datasets and number of overlapping genes and pathways between in vivo and in vitro datasets

Comparison of treatments^a^	Species (age)^b^	Vitro concentration or dilution-factor diet (Rye)	Vivo concentration or dilution-factor diet (Rye)	Vivo^d^ day	Vivo^e^	Vitro^e^ 2h/6h	Genes overlap 2h/6h	Pathways overlap 2h/6h
ZnO vs.Control^a^	pig (weaned)	0.03125% w/v	0.25% w/w	23	101_36^c^	174/720	4_1^c^/3_3^c^	7_1^c^/2_0c
				35	11_11^c^		0_1^c^/2_1^c^	2_3^c^/2_4^c^
Amoxicillin vs.Control^a^	Broiler (1d)	0.5% w/v	0.0067% w/v	5	51	87/705	0/2	1/1
				14	36		0/1	2/2
rye 10% vs. 0%^a^	Broiler (1d)	3	1	21	238	568/21	9/1	1/1
				28	219		8/0	3/3
rye 5% vs. 0%^a^	Broiler (1d)	3	1	21	318	204/26	9/1	4/2
				28	34		1/0	0/0
rye 10% vs.5%	Broiler (1d)	3	1	21	47	455/17	0/0	1/0
				28	192		6/0	4/0

^a^For comparisons with IPEC-J2 cells, the ‘control’ (ZnO and amoxicillin) or ‘0%’ (rye) is a culture medium without additive or rye diet (except for the 10% rye vs. 5% rye comparison). For the ZnO in vivo experiment, the ‘contol’ is a regular diet with a normal level of Zn (60–100 mg/kg)

^b^Age of broilers at the start of the rye and amoxicillin interventions and age of piglets (weaned) at the start of the animal experiment. Piglets were fed with a diet with a higher dose of zinc oxide (2500 mg/kg) from days 14 to 23 post-weaning

^c^Number of genes in the jejunum and number of genes in the ileum

^d^Number of days of sampling of intestinal tissue

^e^Number of DEGs with a *p* value <0.05 and absolute fold change of >2.0 or <0.5 in vitro and in vivo

### IPEC-J2 in vitro test

IPEC-J2 cells were seeded in 2 cm^2^ tissue culture wells (M24 plate) and grown for 7 days at 37 °C and 5% CO_2_ using 1:1 DMEM/Ham’s F10 1:1 medium (Gibco-BRL) supplemented with 5% FCS without antibiotics. For all tests, confluent monolayers were washed twice with medium without FCS (hereafter denoted as medium) and incubated for 1 h with this medium. Hereafter, the medium was discarded and an additive dissolved/suspended in medium was added. Concentrations of 2.5% *w*/*v* amoxicillin (brand name Octacillin) and 0.03125% *w*/*v* of ZnO and of a threefold diluted suspension of the diets containing 5 or 10% *w*/*v* rye in culture medium were used for incubation of IPEC-J2 monolayers for a period of 2 and 6 h. Note that the concentration of amoxicillin used for IPEC-J2 cells was much higher compared to that used in vivo, 0.5 and 0.0067 w/v%, respectively. IPEC-J2 cells were cloned under antibiotic pressure, making them insensitive to exposure to amoxicillin concentrations normally used in cell culture. Therefore, it was necessary to expose IPECJ2 to a high concentration to provoke a response. At these concentrations, no microscopic visible changes to the morphology of the cells and the integrity of the IPEC-J2 monolayers were observed after incubation for 8 h. All incubations were tested in duplicate, and for each type of intervention, duplicate control wells containing no intervention (only culture medium for ZnO and amoxicillin) or the threefold diluted control diet without rye (0% rye) were incubated on the same culture plate for the same period. After incubation for 2 or 6 h, total RNA was extracted from cells using Trizol (Invitrogen). All the RNA samples scored a RNA integrity number (RIN value) of ≥9 (Agilent Lab-on-a-Chip Bioanalyzer). Duplicate RNA samples of duplicate incubations were hybridised separately on a microarray patch (not pooled).

### Microarrays, labelling, hybridization, scanning and selection of DEGs

Custom-prepared 8x60K Agilent pig microarrays G2519F *Sus scrofa* (035953; V2026440) containing 43,803 probes representing 28,369 annotated pig messenger RNA (mRNAs)/genes [51] were used for single dye hybridizations with Cy3-labelled cRNA. Labelling, hybridization, scanning and feature extraction was performed as described previously [51] with minor differences. In Additional file 1, a description of these procedures is described. The raw microarray data are available at the Gene Expression Omnibus (GEO; ncbi.nlm.nih.gov/geo/) under accession numbers GSE67452 for amoxicillin, GSE94095 for rye, GSE94370 for zinc and GSE94139 for IPEC-J2.

DEGs with a fold change (FC) of 2.0 (upregulation) or 0.5 (downregulation) in a microarray comparison of two treatments were selected for further analysis. For each IPEC-J2 comparison of two treatments, a list of DEGs was prepared containing information about the FC, its regulations (up or down) and its gene symbol in Additional file 2: Table S2. List of DEGs of the in vivo experiments was retrieved from the data in the reports published online [32, 50, 57] and listed along with the in vitro data in this Additional file 2: Table S2. For each intervention tested, a list of overlapping DEGs, i.e. DEGs present in lists of both the in vivo and in vitro datasets, was extracted. Throughout this manuscript and in the supplementary files, official human gene symbols (HUGO Gene Nomenclature Committee: http://www.genenames.org) were used in the text and in all figures, tables and appendixes.

### Bioinformatics analysis and data mining

In the results and discussion sections information about the biological function of genes was retrieved by consulting the ‘GeneCards’ (Weizmann Institute of Science) and the NCBI Gene reports (Entrez). The ‘GeneAnalytics’ programme [LifeMap Sciences, Inc] was used to assign genes to a specific pathway, and the Database for Annotation, Visualization and Integrated Discovery (DAVID version 6.7) was used to retrieve enriched Gene Ontology terms (GO-term) with a *p* value <0.05. [16]. Additional HUGO gene symbols of all DEGs (up- and downregulated) from lists of each comparison were loaded in the programme. Because far more human genes are annotated and more information in databases is available for humans than for pigs, a human background was used for this functional analysis. From GeneAnalytics output files, pathways were retrieved with a high or medium enrichment score (*p* value <0.05). Results obtained from GeneAnalytics were categorised and compared between the in vivo and in vitro experiments. The MAPK/ERK signalling pathway was excluded because this pathway is constitutively active in almost every type of cell. The remaining pathways and processes of interest were further investigated in relation to the different dietary interventions. DEGs within each IPEC-J2 datasets coding for secreted effector proteins (e.g. cytokines/chemokines, growth factors and inhibitors) and for enzymes that metabolise chemical effector molecules (leukotrienes), or important inhibitors or stimulators of defined transcriptional mechanisms of in vivo pathways (e.g. mTor), were selected using the genotyping programme VarElect (LifeMap Sciences, Inc.).

## Results

### Correlation between in vivo and in vitro gene expression data

Each microarray comparison between an intervention and control treatment was analysed separately to extract DEGs with a cut-off of *p* value <0.05 and an absolute fold change (FC) of >2.0 up- or downregulated from all in vitro and in vivo datasets (Table 1). In Additional file 2: Table S2, lists of DEGs (as HUGO gene symbol) detected in all comparisons with their direction of regulation (up or down) and with FC are provided. For ZnO and amoxicillin, relatively more DEGs were detected in IPEC-J2 bioassay than in mucosal scrapings. The number of DEGs detected at 6 h was higher than at 2 h in the ZnO and amoxicillin in vitro determined datasets. In contrast to ZnO and amoxicillin, for rye-based diets, significantly less DEGs were detected in the IPEC-J2 dataset of 6 h than in that of 2 h, indicating that up- or downregulated expression of the majority of genes at 2 h was normalised at 6 h. Only a limited number of overlapping genes were present in in vivo and in vitro lists of DEGs, most of them in the datasets of rye. In Additional file 2: Table S2, all overlapping DEG genes of in vitro and in vivo comparisons are listed for each additive with their full names. Overlapping genes discussed in the text of this manuscript are also listed in Table 3 with a brief description of their function (see below).

The number of overlapping pathways/GO-terms between in vitro and in vivo interventions extracted from output files generated by the bioinformatics programme GeneAnalytics was also limited for the three ingredients. Also, no correlation could be found between the number of overlapping DEGS and overlapping pathways/GO-terms for all three interventions. Overlapping pathways/processes and related non-overlapping in vivo pathways/processes are listed in Table 2. DEGs overlapping between in vitro and in vivo interventions and participating in these pathways/processes or GO-terms are underlined in the ‘response genes’ column in Table 2.

**Table 2:** Pathways/processes and GO-terms overlapping between in vitro and in vivo datasets

System/additive	Time	Pathway or Go-term	Score^a^	No. of total genes^b^	No. of DEGs^d^	Response genes^e^
	Overlap Zno					
Ileum/ZnO	23 days	Mineral absorption	15.1	51	4	SLC39A4, MT1F, HEPH, *MT1A*
IPEC-J2/ZnO	2 h	Mineral absorption	20.4	51	5	HMOX1, S100G, SLC30A1, *MT1A*
Jejunum/ZnO	23 days	PAK pathway	12.7	620	9	CDH2, PLA2G7, *FOS*, CXCL13, MYLK, PAK1, OSM, TUBA8, GDF6
IPEC-J2/ZnO	2 h	PAK pathway	15.0	731	18	BMP2, CSF2, CXCL2, *FOS*, GDF15, HBEGF, IL1A, IL6, IL8, JUN, KLF10, MLLT4, NGF, NRG1, SLC2A4, SNAI1, TCF21, ZYX
Jejunum/ZnO	23 days	Influenza A	10.9	317	6	*FOS*, HSPA8, OAS2, RSAD2, *HLA-DQA1*, DDX58
IPEC-J2/ZnO	2 h	Influenza A	16.3	317	12	DNAJB1, *FOS*, *HLA-DQA1*, HSPA1A, HSPA1B, IL1A, IL6, IL8, JUN, SOCS3, TICAM1, TNFAIP3
Jejunum/ZnO	23 days	Cytoskeleton remodelling keratin filaments	20.9	48	5	KRT7, PLEC, TUBA1C, TUBA8, TUBA3C
Jejunum/ZnO	23 days	Cytoskeletal signalling	13.0	242	6	ARHGAP35, KRT7, MARK1, PLEC, PAK1, DES
Jejunum/ZnO	23 days	Cytoplasmic microtubules	24.5	102	7	DYNC2H1, MARK1, PLEC, TUBA1C, TUBA8, TUBA3C, DES
IPEC-J2/ZnO	2 h	Cell adhesion_ECM remodelling	9.3	61	4	HBEGF, IL8, PLAU, SERPINE1
Jejunum/ZnO	23 days	Cell adhesion molecules (CAMs)	9.7	145	4	CD8A, CDH2, VTCN1, *HLA-DQA1*
Jejunum/ZnO	23 days	Cell adhesion gap junctions	10.9	49	3	TUBA1C, TUBA8, TUBA3C
IPEC-J2/ZnO	6 h	Adhesion	11.0	80	10	ADAM9, CLDN1, CTNNB1, CYR61, DOCK1, ITGA5, MPZL1, NEDD9, NOV, SERPINE1
	Overlap rye					
IPEC-J2 10 vs. 5%/rye	2 h	Cell cycle	11.1	538	24	ATM, CASC5, CDC27, CENPF, CEP152, CNTRL, KIF20A, LMNA, MAD1L1, NINL, NIPBL, PCM1, PDS5B, PSMB4, RPS27A, SGOL2, SMC2, SMC3, SMC4, STAG2, SYNE2, TOP2A, TPR, UBB
Jejunum 10%/rye	28 days	Cell cycle	10.1	538	11	ARPP19, OPTN, RAB1A, MAD2L1, FZR1, RFWD2, E2F4, NUP35, NUP214, YWHAG, RUVBL1
IPEC-J2 10%/rye	2 h	Cell cycle	8.3	538	4	ATM, CENPF, NIPBL, SYNE2
Jejunum 5%/rye	21 days	Cell cycle	15.2	538	17	ARPP19, CDK2, OPTN, RAB1A, CSNK2B, MAD2L1, CNTRL, PSMD11, CENPE, GINS2, E2F2, MCM10, SGOL1, NUP160, NUP214, SMC1B, RUVBL1
Jejunum 10%/rye	28 days	Cell cycle/checkpoint control	7.9	229	6	ARPP19, MAD2L1, VCP, YWHAG, MKI67, ZBTB17
Jejunum 10 vs. 5%/rye	28 days	Cell cycle/checkpoint control	8.7	229	6	ARPP19, MAD2L1, VCP, MKI67, MLH1, ZBTB17
IPEC-J2 10 vs. 5%/rye	2 h	Cell cycle/checkpoint control	13.1	229	15	ATM, CDC27, CEND1, FOSB, LMNA, NHEJ1, PCM1, RAD50, RAN, SMC2, SMC3, SMC4, SMG1, STAG2, TPT1
IPEC-J2 10 vs. 5%/rye	2 h	Biosynthesis of the *N*-glycan precursor (dolichol–LLO) and transfer to a nascent protein	46.3	696	51	ACTB, EEF1A1, EEF1A2, EEF1G, FAU, GFPT1, LMAN1, LMNA, MBOAT4, *MUC4*, SLC25A6, SLC30A7, TUBA1B, *RPL19*, *RPL5*, including 36 RPL and RPS variants
Jejunum 10 vs. 5%/rye	28 days	Biosynthesis of the *N*-glycan precursor (dolichol–LLO) and transfer to a nascent protein	6.9	696	10	ALG14, COQ2, RPL37, SRP9, ST3GAL4, LDHD, IGFBP1, PIGA, F2, ZBTB17
Jejunum 5%/rye	21 days	Biosynthesis of the *N*-glycan precursor (dolichol–LLO) and transfer to a nascent protein	15.6	696	20	ARFGAP1, CTSG, GCNT3, EIF4B, ALG14, COQ2, RPL37, *RPL19*, *RPL5*, SRP9, ST3GAL4, LDHD, *MUC17*, PFDN1, TPP1, INHA, IGFBP1, ATP6AP2, SPHK1, F2
IPEC-J2 5%/rye	2 h	Biosynthesis of the *N*-glycan precursor (dolichol–LLO) and transfer to a nascent protein	87.5	696	39	ACTB, EEF1A1, EEF1G, *MUC13*, STS, TUBA1B, *RPL19*, *RPL5*, including 32 RPL and RPS variants
	Rye; related in vivo pathways					
Jejunum 10 vs. 5%/rye	28 days	Signalling by BMP	6.8	48	3	NUP214, TOB1, *ZFYVE16*
Jejunum 10 vs. 5%/rye	21 days	Ovarian infertility genes	21.0	32	4	ESR2, INHA, NR5A1, ZP3
Jejunum 10 vs. 5%/rye	21 days	Ca-dependent events	9.6	247	4	CYP11A1, NR5A1, OPRM1, STAR
	Overlap amoxicillin					
Jejunum/amox	14 days	CC_FAT/GO:0015630 ~ microtubule cytoskeleton	0.016	31^c^	5	IFT20, *MID1IP1*, STMN1, MAP7D1, TACC1
IPEC-J2/amox	6 h	CC_FAT/GO:0015630 ~ microtubule cytoskeleton	0.241	707^c^	21	APC2, NIN, IFT80, FLOT1, ALMS1, *MID1IP1*, MID1, MARK1, TUBGCP3, KIF1B, RCC2, DYNLL1, CEP350, CLIC5, KIFAP3, TBCC, TUBA4A, STRBP, NDRG2, KATNAL1, TUBB3
Jejunum/amox	14 days	CC_FAT/GO:0005856 ~ cytoskeleton	0.009	31^c^	8	ARPC1A, IFT20, MYL12A, *MID1IP1*, STMN1, HNRNPH1, MAP7D1, TACC1
	Amoxicillin; related in vivo pathways					
Jejunum/amox	14 days	Aurora B signalling	10.2	39	2	TACC1, STMN1
Jejunum/amox	6 h	Validated targets of C-MYC transcriptional repression	17.9	62	11	CCND1, CDKN1B, CEBPD, CLU, DDIT3, DKK1, NDRG1, NDRG2, RBL1, S100A7, *ZBTB17*
Jejunum/amox	6 h	Cell cycle/checkpoint control	11.9	229	18	BRD2, CCND1, CDC25A, CDKN1B, CSNK1A1, DDIT4, FOSB, KAT5, NBN, NEK7, PLK3, RBBP8, RCC2, SMG1, TFAP2C, TP53BP1, YWHAG, *ZBTB17*

^a^GeneAnalytics pathways with a high score (>18; corresponding with an FDR-corrected *p* value ≤0.0001) and medium score (>6.5; corresponding with an FDR-corrected *p* value ≤0.1) were retrieved. Enriched GO-terms with a *p* value of <0.05 were retrieved from DAVID, except for IPEC-J2 cells at 6 h. GO-terms with a *p* value of <0.25 were retrieved. Pathway annotations are from GeneAnalytics (http://geneanalytics.genecards.org).

^b^Number of genes; total number of genes in a pathway or number of DEGs loaded in DAVID (for GO-term analysis)

^c^Number of DEGs loaded in DAVID (for GO-term analysis)

^d^Number of DEGs mapped to a pathway or to a GO-term

^e^Overlapping genes are italicized. Pathway annotations are from GeneAnalytics (http://geneanalytics.genecards.org).

### Selection of early ‘effector genes’ from IPEC-J2 datasets

Gene enrichment analysis using the bioinformatics programme GeneAnalytics showed that the in vivo processes of ‘mineral absorption’, ‘cell adherence and tight junction formation’ and ‘antiviral response’ for ZnO, ‘microtubule and cytoskeleton integrity’ for amoxicillin and ‘cell cycle progression and mucus production’ for rye were significantly enriched. These processes are modulated in the intestinal mucosa by these dietary interventions. We therefore extracted a set of effector genes differentially expressed in the enterocyte cell line IPEC-J2 cells that have the potential to affect biological processes in the intestine. For selection, the terms ‘secreted’, ‘growth factor’, ‘cytokine’, ‘chemokine’, and ‘leukotriene’ were used to extract these ‘effector genes’ from lists with DEGs. In addition, information about the function of these putative ‘effector genes’ in databases was consulted to review their regulatory/steering role in the intestine and in the overlapping (or related) pathways and GO-terms mentioned above. In Table 3, these selected ‘effector genes’ are listed together with the DEGs that overlapped between the in vivo and in vivo datasets and were mapped to an overlapping pathway/process. In Additional file 2: Table S2, these ‘effector genes’, with their FC, are highlighted in red. In the text below, all these IPEC-J2 ‘effector/regulators’ are marked with a superscript capital ‘E’ (e.g. MT1A^*E*^). Note that a few of the overlapping DEGs were also identified as a putative ‘effector gene’ and are also marked with ‘E’ in the text below.

**Table 3:** Overlapping in vivo and in vitro DEGs and selected IPEC-J2 effector genes from ZnO, rye and amoxicillin datasets

Gene symbol	Full name	Day in vivo/hours in vitro IPEC-J2	Function/part of process
	ZnO overlapping genes		
ZFP36L2	ZFP36 ring finger protein-like 2	23 days/2 h	Promotion mRNAs deadenylation and degradation
MT1A	Metallothionein 1A	23 and 35 days-ileum-23 days-jej/2 and 6 h	Divalent metal ion transporter and scavenger of oxygen radicals
	ZnO; putative IPEC-J2 effector genes	Related or overlapping in vivo pathway	
HBEGF	HBEGF (heparin-binding EGF-like growth factor)	PAK pathway	Mitogenic for fibroblasts multiple organ systems
NRG1	NRG1 (neuregulin 1)	PAK pathway	Growth and development of multiple organ systems
BMP2	BMP2 (bone morphogenetic protein 2)	PAK pathway	Member TGF-beta superfamily/induces bone and cartilage formation
NGF	Nerve growth factor	PAK pathway	Nerve growth-stimulating activity
MT1A	MT1A (metallothionein 1A)	Mineral absorption	Divalent metal ion transporter and scavenger of oxygen radicals
HMOX1	Heme oxygenase 1	Mineral absorption	Enzyme heme catabolism/response to hypoxya/Fe^2+^ transport/regulation of vascular tone
SERPINE1	SERPINE1 (serpin family E member 1)	Cell adhesion_ECM remodelling	Serine protease inhibitor/inhibitor of fibrinolysis
IL6	Interleukin 6	Influenza A/PAK pathway	Cytokine that induces inflammation and maturation of B cells
IFNL1	Interferon λ1	Influenza A/PAK pathway	Antiviral host defence in the epithelial tissues
IL1A	Interleukin 1A	Influenza A/PAK pathway	Pleiotropic cytokine/role in various immune responses and inflammation/antiviral response
IL8	(Interleukin 8)	Influenza A/PAK pathway	Chemotactic factor attracting neutrophils, basophils, and T cells
IL21	(Interleukin 21)	None	Cytokine promoting switch between innate and adaptive immunity
	Rye overlapping genes		
HOOK3	Hook microtubule-tethering protein 3	10% 21 days, 10 vs. 5% 28 days, 10% 28 days/2 h	Component FTS/Hook/FHIP complex/vesicle trafficking
PTEN	Phosphatase and tensin Homolog	10% 21 days, 10 vs. 5% 28 days, 10% 28 days/2 h	Protein tyrosine Phosphatases/negatively regulating AKT-PKB signalling
ZFYVE16	Zinc finger, FYVE domain-containing 16	10% 21 days, 10 vs. 5% 28d/2 h	Recruits TGF transcriptional modulators/membrane trafficking endosomes
CCK	Cholecystokinin	10% 21 days, 10 vs. 5% 28d/2 h	Peptide hormone induces release of pancreatic enzymes
NOG	Noggin	5% 21 days/2 h	Inactivates members of TGF-beta family signalling proteins (e.g BMPs)
MUC(17)	Mucin 17, cell surface-associated	5%-21d, 10% vs. 5% 21d/6 h	Membrane-bound mucin that provides protection to gut epithelial cells
	Rye; putative IPEC-J2 effector genes	Related or overlapping in vivo pathway	
TNF	Tumour necrosis factor	TGF-beta signalling	Multifunctional proinflammatory cytokine
FST	Follistatin (activin-binding protein)	TGF-beta signalling	Inhibits follicle-stimulating hormone release/antagonist of INHBA
RICTOR	RPTOR independent companion of MTOR complex 2	mTOR signalling	Subunit of mTORC2/regulates cell growth and survival in response to hormones
DDIT4	DNA damage-inducible transcript 4	mTOR signalling	Regulation cell growth and survival/inhibition activity mTORC1 in response of hypoxia
MUC(4)	Mucin 4, cell surface-associated	*N*- and *O*-linked glycan synthesis	Sialomucin/intestinal epithelial cell differentiation/EGF cell signalling
MUC(13)	Mucin 13, cell surface Associated	*N*- and *O*-linked glycan synthesis	Epithelial and hemopoietic transmembrane mucin/EGF cell signalling
B3GALT1	Beta-1,3-galactosyltransferase 1	*N*- and *O*-linked glycan synthesis	Transfers galactose from UDP galactose to a terminal beta-*N*-acetylglucosamine
	Amoxicillin-overlapping genes		
OSER1	Oxidative stress responsive serine-rich 1	5/2 h	Cellular response to hydrogen peroxide/UBC binding
ZBTB17	Zinc finger and BTB domain-containing 17	5/6 h	c-MYC transcriptional repression (regulation of cell cyle)
MID1IP1	MID1-interacting protein 1	5/6 h	Negative regulation of microtubule (de)polymerisation/UBC binding
STMN1	Stathmin 1^a^	5 and 14/	Prevents assembly and promotes disassembly of microtubules/UBC binding
EREG	Epiregulin^a^	5 and 14/	Member of the epidermal growth factor family/UBC binding
CLTA	Clathrin, light chain A^a^	5 and 14/	Coated pits and vesicles and receptor-mediated endocytosis/UBC binding
ARPC1A	Actin-related protein 2/3 complex, subunit 1A^a^	5 and 14/	Actin polymerisation-forming of filopodia or stress fibres/UBC binding
	Amoxicillin; putative IPEC-J2 effector genes	Related or overlapping in vivo pathway	
HMOX1	Heme oxygenase 1	HIF1A pathway	Essential enzyme heme catabolism/response to hypoxia/regulation of vascular tone
HK2	Hexokinase 2	HIF1A pathway	Phosphorylate glucose to produce glucose-6-phosphate
PDK1	Pyruvate dehydrogenase kinase 1	HIF1A pathway	Regulation of glucose and fatty acid metabolism/upregulated in response to hypoxia
EDN1	Endothelin 1	HIF1A pathway	Potent vasoconstrictor
NPPA	Natriuretic peptide A	HIF1A pathway	Regulation blood pressure and body fluid homeostasis
MID1	Midline 1 RING finger protein	Microtubule	E3 ubiquitin ligase/microtubule (de)polymerisation/UBC binding

^a^In addition to overlapping genes for amoxicillin information of DEGs in the in vivo dataset related to overlapping pathways and regulated at both time points (5 and 14 days) is provided

### Association of IPEC-J2 ‘effector genes’ with overlapping pathways/processes

For each of the interventions below, we provided information from biological databases and related literature about the function of the selected ‘effector genes’ associated to the identified overlapping pathways/processes. For fold changes (FC) and up- or downregulation of DEGs, we refer to Additional file 2: Table S2.

#### ZnO effector genes

**Fig. 1:**
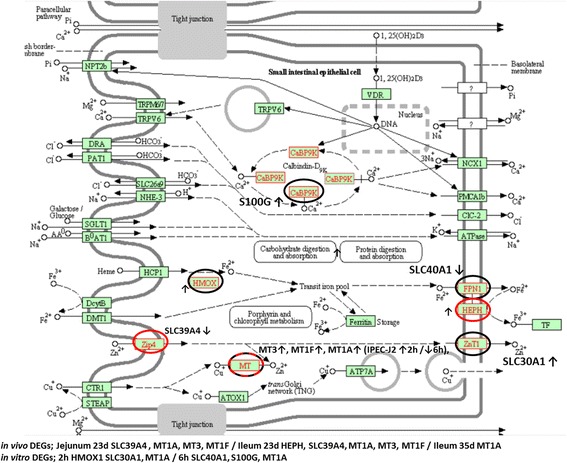
KEGG mineral absorption pathway. Genes specifically responding to zinc in the jejunum or ileum of piglets are encircled in *red* and in IPEC-J2 cells in *black*. MT1A gene expression was upregulated in the ileum and jejunum of piglets, in IPEC-J2 cells at 2 h, and downregulated in IPEC-J2 cells at 6 h. *Arrows* indicate up (↑)- or down (↓)-regulation. Official gene symbols are provided for non-HUGO symbols used in the boxes of the pathway (FPN1 = SLC40A1, ZnT1 = SLC30A1, Zip1 = SLC39A4, CaBP9K = S100G)

The overlapping genes responding to zinc oxide in the in vitro and in vivo datasets were limited to nine unique DEGs from which three were part of an overlapping pathway (Additional file 3: Table S3). Among the overlapping genes was the effector metallothionein 1A (MT1A^*E*^), a divalent metal ion transporter. MT1A^*E*^ was strongly upregulated (38-fold) in the jejunum at 23 days (9 days after the start of the ZnO intervention) and in IPEC-J2 cells (tenfold). Several other genes involved in regulation of divalent metal ion homeostasis in cells were also differentially expressed in response to ZnO. We detected regulation of two other MT variants (MT-III and MT1F in piglets), several membrane transporters for Zn^2+^ and Fe^2+^ (SLC39A4, HEPH and SLC40A1 in piglets and SLC30A1 and HMOX1^*E*^ in IPEC-J2 cells) and the intracellular Ca^2+^ transporter S100G (in IPEC-J2 cells). In Fig. 1, all these DEGs are marked in the KEGG scheme of ‘mineral absorption pathway’. MTs function also as potent scavengers of reactive oxygen species (ROS) and influence the redox/oxidative status of cells [48].

Pigs fed with a high ZnO diet showed regulation at 23 days in the jejunum of a relatively high percentage of genes (20 out of the 101 total DEGs) involved in cell adherence/adhesion and cytoskeleton remodelling/signalling. In addition, the PAK signalling pathway, a signalling cascade involved in regulation of structural processes in cells and tissues (epithelial cells included) overlapped between the jejunum at 23 days and IPEC-J2 cells at 2 h, and the PAK1 gene (alias p21 protein or Cdc42/Rac-activated kinase 1) itself were differentially expressed at 23 days in the jejunum of piglets. Four growth factors, part of the PAK signalling pathway (HEBGF^*E*^, NRG1^*E*^, NGF^*E*^ and BMP2^*E*^), were found to be upregulated in IPEC-J2 cells in response to ZnO at 2 h. These growth factors bind to TGF, EGF/EBR and FGF surface receptors involved in steering adhesion/adherence and tight junction processes. In Additional file 4: Table S4, all these genes (S4a) regulated in both the jejunum at 23 days and in IPEC-J2 cells at 2 and 6 h and related to cell adherence/adhesion and cytoskeleton remodelling/signalling are listed and mapped to pathways (S4b) using GeneAnalytics analysis.

Bioinformatics analysis of the in vitro ZnO dataset indicated that MT1A^*E*^ is a functional component of a mechanism by which IPEC-J2 cells respond to oxidative stress [31]. Together with the cytokine IL6^*E*^, MT1A^*E*^ it is an important regulator of hypoxia-induced factor 1 (HIF1)-mediated transcription of effector molecules (e.g. HMOX1^*E*^ and SERPINE1^*E*^) involved in restoring normoxia in enterocytes ([31] and references herein). In contrast to IL6^*E*^, for which we observed downregulated gene expression at 2 h, expression of several other cytokines (IL8^*E*^, IL21^*E*^, IFNL1^*E*^ and IL1A^*E*^) was upregulated in IPEC-J2 cells at 2 h. Similar as observed for IL6^*E*^, gene expression of these cytokines was normalised or even reversed at 6 h, indicating a rapid decay of their mRNAs. Only expression of the gene coding for IL21^*E*^, a cytokine promoting the switch between innate and adaptive immunity and involved in production of IgA-producing B cells in the intestine, was found to be upregulated at 6 h. In IPEC-J2 cells at 2 h, as well as in the jejunum at 23 days, expression of the ZFP36L2^*E*^ gene (alias butyrate response factor 2/EGF-response factor 2) was regulated in response to zinc. ZFP36 gene variants code for early response proteins that bind to AU-rich elements in the 3′ region of mRNAs and recruit exosomes and enzymes to degrade mRNAs [49].

An overlap was found for the ‘influenza virus’ pathway and included the regulation of the antiviral genes 2′,5′-oligoadenylates transferase (OAS2), viperin (RSAD2) and DEXD/H-box helicase 58 (DDX58; alias RIG1). Gene expression of the IFNL1^*E*^ and IL1A^*E*^, cytokines able to activate a dsRNA-mediated antiviral response, were upregulated at 2 h.

#### Rye effector genes

Cell cycle/mitosis and synthesis of *N*-glycan precursors were identified as overlapping pathways/processes between the IPEC-J2 dataset at 2 h and in vivo datasets obtained at 21 and 28 days in broilers (Table 3). At 21 days, gene expression of mucin 17 (MUC17) was upregulated in the jejunum of the broilers. In IPEC-J2 cells, two paralogs of this gene, MUC4^*E*^ and MUC13^*E*^, were strongly downregulated at 2 and 6 h. All these three mucins are highly glycosylated proteins anchored in the apical membrane of the epithelial cells forming the so-called glycocalyx layer, a mucus layer on top of the epithelial cells [35]. Besides their barrier function in preventing invasion of pathogenic bacteria, mucins contain a membrane-anchored EGF-like domain that transmits signals to the interior of the cell [28].

**Fig. 2:**
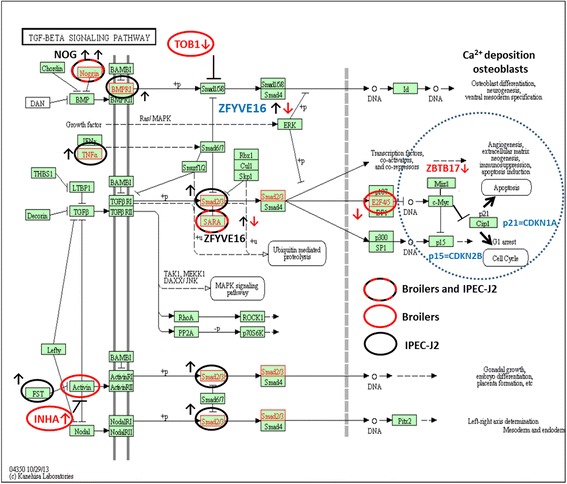
KEGG TGF-beta signalling pathway. Genes specifically responding to rye in the jejunum of broilers are encircled in *red* and in IPEC-J2 cells in *black*. Overlapping genes ZFYVE16 and NOG are encircled by *dashed red-black lines*. The DEG ZBTB17 (alias MIZ1) and gene CDKNA1 (alias p21) were also part of the KEGG cell cycle pathway and integrated in the original scheme of the KEGG TGF-beta signalling pathway (encircled with a *dashed line*) and are discussed in the section of amoxicillin. *Arrows* indicate up (↑)- or down (↓)-regulation. Official gene symbols are provided for non-HUGO symbols used in the boxes of the pathway scheme (NOG = Noggin, SARA = ZFYVE16)

Bioinformatics analysis of sets of DEGs from the in vivo rye study revealed regulation of the TGF-beta (depicted in Fig. 2) and mTOR signalling pathways [57], with a pivotal role for the genes inhibin A (INHA, a member of the TGF-beta superfamily) and the overlapping gene PTEN^*E*^ (phosphatase and tensin homolog), respectively. PTEN^*E*^ was also regulated in IPEC-J2 cells at 2 h. PTEN^*E*^ is a key modulator of the (PKB)-AKT/mTOR signalling, a pathway that regulates processes like cell proliferation, adhesion, migration, invasion, apoptosis and translation. With respect to AKT/mTOR signalling, gene expression of the mitochondrial hexokinase HOOK3 was regulated by the 10% rye diets in broilers and in IPEC-J2 cells. HOOK3 is also part of the AKTIP (FTS)/Hook/FHIP complex that directly enhances the kinase activity of (PKB)/Akt [61]. In IPEC-J2 cells, expression of the gene RICTOR^*E*^, a functional component of the mTOR complex increased after 2 h, and expression of the mTOR-inhibitor DNA damage-inducible transcript 4 (DDIT4^*E*^), vigorously decreased (>40-fold) within 6 h.

The INHA gene is coding for a preproprotein cleaved to the alpha subunit of inhibins A and B (INHBA an INHBB). Different homo- and heterodimer complexes of these inhibins activate or inhibit signalling via the BMP, TGF-beta and activin receptors resulting in activation or inhibition of cell division and proliferation, including that of stem cells. An antagonist of inhibins, follistatin (FST^*E*^; activin-binding protein), was downregulated in IPEC-J2 cells. Several other regulators/modulators of TGF-beta signalling were differentially expressed in broilers and IPEC-J2 cells in response to rye. Among them were the overlapping DEGs noggin (NOG^*E*^) and endofin (ZFYVE16^*E*^; alias SARA) and ZBTB17 (alias MIZ). For ZBTB17, no regulated gene expression was observed in IPEC-J2 cells. ZFYVE16^*E*^ is a scaffold protein that recruits the TGF transcriptional modulators SMAD2 and 3 to the TGF-beta receptor. ZBTB17 interacts with MYC resulting abolishment of the repression by MYC of the transcription of cell cycle inhibitors like CDKN2B (alias p15) and CDKN1A (alias p21). TGF-beta-mediated arrest of epithelial cell division is executed by CDKN2B, and therefore, downregulation of ZBTB17 in broilers may stimulate cell division [60]. The cytokine TNF^*E*^ (upregulated by 10% rye at 2 h in IPEC-J2 cells) may inhibit signalling induced by binding of TGF-beta to its receptor. The effect of TNF^*E*^ on downstream processes induced by TGF-beta signalling can be diverse. In a scheme of TGF-beta signalling (Fig. 2), all above and beneath mentioned DEGs involved in transcriptional regulation of cell cycle stimulation/inhibition are depicted.

The scaffold protein ZFYVE16^*E*^ functions also as anchor for bone morphogenetic protein (BMP) receptors. Gene expression of NOG^*E*^ and TOB1 (transducer of ErbB2,1), both extracellular inhibitors of BMP-SMAD-mediated transcription (Fig. 2), was found down- and upregulated in response to 5% rye diet at 21 and 28 days, respectively. Gene expression in IPEC-J2 of NOG^*E*^ and the BMP-receptor BMPR1A cells was upregulated by 5% and 10% rye diets. Inhibition by NOG^*E*^ and TOB1 negatively regulates BMP-SMAD-mediated transcription of genes that stimulate proliferation of osteoblast cells, cells responsible for calcium deposition and bone formation (see Fig. 2). It was shown that FST^*E*^ antagonises signalling by BMPs and affects Ca^2+^ deposition [21].

For the in vivo comparison 10 vs. 5% rye at 21 days, the pathways ‘calcium-related events’ and ‘steroid hormone synthesis’ in ovarian granulose cells (‘ovarian infertility’ pathway; http://www.wikipathways.org/index.php/Pathway:WP34) and ‘BMP signalling’ were called significant (see Table 3). Two DEGs coding for ‘zona pellucida’ glycoproteins (ZP3 and uromodulin, alias ZPD) showed a highly variable, rye concentration-dependent level of gene expression at days 21 and 28 in broilers. Like mucins, ZP glycoproteins are highly *N*- and *O*-glycosylated proteins. They form extracellular filament-like structures on the surface of vertebrate oocytes. In IPEC-J2 cells, a high and concentration-dependent upregulation (~40-fold in 10% rye comparison; Table S2) of beta-1,3-galactosyltransferase 1 (B3GALT1^*E*^) gene expression was induced. At the surface of oocyte, BGALT enzymes interact with ZP glycoproteins to form a complex facilitating interaction between the oocyte and sperm cell. For the overlapping gene coding for the peptide gut hormone cholecystokinin (CCK^*E*^, a hunger suppressant), no relevant association was found to the overlapping processes of cell cycle and *N*-glycan synthesis. Cell surface receptors for CCKA and B belong to the large family of G protein-coupled receptors, from which many, including CCKA and B, can be involved in modulation of the intracellular calcium level [59].

#### Amoxicillin effector genes

In the in vivo dataset of amoxicillin at 14 days, 16% of the DEGs were associated with the GO-term ‘microtubule cytoskeleton’. Together with actin filaments, microtubule filaments (MTub) determine the morphology and internal structural organisation of cells. They are the backbone for centromere spindles in the nucleus during cell division. The genes ‘transforming acidic coiled coil-containing protein 1’ (TACC1), stathmin 1 (STMN1) and overlapping gene MID1IP1^*E*^, all downregulated at day 14 in broilers, are important players in the depolymerisation process of microtubule (MTub) filaments. Both TACC1 and STMN1 interact with the Aurora B kinase, a kinase that inhibits microtubule (MTub) depolymerisation at the negative ends of filaments. In addition, MID1IP1 binds to the E3 ubiquitin-protein ligase midline-1 (MID1^*E*^). In IPEC-J2 cells, both genes were upregulated at 6 h. In the (de)polymerisation process, MID1^*E*^ anchors microtubules to the cytoskeleton. Many DEGs in the in vivo dataset that code for MTub-associated proteins also conjugate with Ubiquitin C (UBC). Conjugation with UBC targets proteins for proteasomal degradation. From the IPEC-J2 dataset at 6 h, 21 DEGs functionally associated with the GO-term ‘CC_FAT microtubule cytoskeleton’, however, scoring a *p* value of 0.24 (Table 2). Among these DEGs, several genes are involved in regulation of MTub polymerisation and formation of specific filament structures (TBCC, TUBA4A, TUBB3, MID1^*E*^, MID1IP1^*E*^, MARK1, NIN, ALMS1, CLIC5, TUBGCP3, KATNAL1), lipid rafts (FLOT1) and genes that are essential for motility along the intracellular filaments and sweeping of extracellular protruding cilia (DYNLL1, IFT80, KIFAP3, STRBP and KIF1B). The gene clathrin light chain A (CTLA), regulated at 5 and 14 days in broilers, is involved in vesicle transport along the microtubule filaments in the cytoplasm of the cell.

In IPEC-J2 cells at 2 h and in the jejunum of broilers at 5 days, expression of the gene ‘oxidative stress’ serine-rich 1 (OSER1; alias Perit1) was up- and downregulated, respectively. Expression of OSER1 is induced in cardiac myocytes exposed to H_2_O_2_, and this gene is considered as a biomarker for oxidative stress [10]. One of the main processes induced by amoxicillin in IPEC-J2 cells was oxidative stress. IPEC-J2 cells reacted to hypoxia by HIF1A-mediated transcription of a set of effector genes able to restore oxygen balance and aerobic energy metabolism in these cells [31]. Expression of HIF1A effector genes HMOX1^*E*^ and NPPA^*E*^, both vasodilators [17, 27], was highly stimulated, and expression of EDN1^*E*^, a vasoconstrictor, was suppressed by amoxicillin at 6 h in IPEC-J2 cells. In addition, expression of genes coding for the sugar-metabolising enzymes HK2^*E*^ and PDK1^*E*^, both promoting anaerobic energy metabolism, was supressed by amoxicillin at 6 h in IPEC-J2 cells.

Binding of growth factors like epiregulin (EREG; downregulated in the jejunum at days 5 and 14) and NRG1^*E*^ (upregulated in IPEC-J2 cells at 2 h) to epidermal growth factor receptor (EGFR)/ErbB receptors steers various processes, which either support cell cycle progression or initiate apoptosis. Expression of Myc, a transcriptional suppressor of cell cycle inhibitor CDKN1A (alias p21; downregulated in IPEC-J2 cells at 6 h), can be activated by EGFR/ErbB signal transduction. Interaction of the overlapping gene ZBTB17 with MYC results in relief of repression of transcription of CDKN1A. In response to DNA damage, this relief stimulates expression of CDKN1A and its anti-apoptotic activity, thereby promoting G1 cell cycle arrest instead of apoptosis [Seoane and Massague 2002]. Involvement of ZBTB17 in Myc transcriptional repression of CDKN1A is implemented in the TGF-beta signalling scheme provided for rye (right side of Fig. 2).

## Discussion

From commercial perspective, long-term effects of dietary interventions are relevant for improvement of animal production. On first sight, the short-term measurement (2 and 6 h) of gene expression in the IPEC-J2 bioassay showed a low predictive value for gene expression that was measured in the intestines of piglets and broilers after feeding them for days/weeks with diets containing the same interventions. Based on the short-term data, however, a set of regulatory and effector genes expressed by enterocytes was identified, which provided more insight on how biological processes imposed by the tested interventions in vivo may be modulated by ‘early’ signalling molecules activated in enterocytes by zinc, amoxicillin or components in the rye diets. Moreover, some of these effector genes also overlapped between in vivo and in vitro and were important components of the overlapping pathways/processes identified in this study, i.e. mineral absorption, cell adherence and tight junction formation for the zinc intervention, microtubule and cytoskeleton integrity for amoxicillin treatment and cell cycle progression for the rye intervention.

### Zinc

Expression of several MT genes, including MT1A^*E*^, was highly upregulated in the jejunum and ileum when a high dose of ZnO was fed to piglets. In IPEC-J2 cells only for MT1A^*E*^, a higher expression was observed and not for the other MT variants. Likely, in response to ZnO, the higher expression of other MTs than MT1A^*E*^ occurred in other cell types than in enterocytes in the intestinal mucosa of pigs. A persistent high expression of MT1A^*E*^ in enterocytes as we observed in IPEC-J2 cells may not only scavenge ROS but may also play a role in regulation of cytokine production (see below) and divalent metal ion absorption. This latter process affects intracellular Ca2^+^ levels in enterocytes and, consequently, cell adherence and tight junction interactions between cells lined up in the epithelial layer [22]. Based on our IPEC-J2 data, it was predicted that these processes may be steered by the growth factors HEBGF^*E*^, NRG1^*E*^, NGF^*E*^ and BMP2^*E*^. This is in agreement with results of several studies in which pigs were fed with a high dose of dietary zinc [43, 52, 53]. These studies showed that surface receptors, to which these growth factors bind, were activated (TGFr, EGF/EBRr and FGFr) in response to high ZnO diet. This activation resulted in restoration of the barrier function and integrity of the intestinal layer. These findings are in line with the observation that ZnO reduced intestinal permeability by stimulating expression of tight junction proteins in weaned piglets [65].

In IPEC-J2 cells, we observed an immediate and short-lasting activation of cytokine gene expression, within 2 h, which normalised or even inverted before 6 h. This is in agreement with an in vivo study in which dietary zinc lowered gene expression of several chemokines, cytokines and inflammatory genes in piglets challenged with ETEC K88, a pathogen causing post-weaning diarrhoea [Sargeant et al. 2010]. Zinc finger proteins are necessary for signal transduction from cytokine receptors to response genes, and MTs are involved in the regulation of this signalling inside the cell [26, 39]. Therefore, it would be interesting to investigate if the ‘overlapping’ zinc finger protein ZFP36L2 binds to the AU-rich elements present in the 3′ region of cytokine mRNAs and recruit exosomes and enzymes to degrade these mRNAs. Such a mechanism induced by zinc may control the half-life of cytokine mRNAs in enterocytes in vivo and could prevent overreaction of the innate immune response.

Interplay between IL6^*E*^ and MT1A^*E*^ was identified as a steering mechanism for HIF1A-mediated transcription of a set of effector genes involved in regulation of oxidative stress in IPEC-J2 cells [31]. A similar interplay was observed in several other in vivo studies [39, 62]. Moreover, it was reported that MT1A^*E*^ expression is under control of IL6^*E*^ [25]. However, only in IPEC-J2 cells we detected a lower level of IL6^*E*^ gene expression, and not in vivo. The persistent high upregulation of MT1A^*E*^ and other variants of MTs, all potent scavengers of ROS, at day 23 in the jejunum and ileum may have maintained, or already restored, normoxia in cells of the mucosal layer, leaving no need to (further) activate HIF1A transcription of effectors molecules.

The differential expression of antiviral genes, like OAS2, RSAD2 and DDX58 (alias RIG1), that we observed in the small intestinal mucosa of piglets was also observed in weaned piglets fed with a high dose of zinc and challenged orally with the RNA virus TGEV, a virus causing diarrhoea in weaned piglets [12]. Together with the higher expression in IPEC-J2 cells of the ‘antiviral’ cytokine genes IFNL1^*E*^ and IL1A^*E*^, this suggested that high levels of ZnO modulate an antiviral response in vivo by promoting expression of these cytokines in enterocytes.

The impact of zinc deficiency and zinc supplementation on human health has been studied extensively in clinical trials (reviewed in [45]). In elderly, zinc supplementation supports the proper functioning of the innate and adaptive immune system [7]. A diet with sufficient zinc and a balanced absorption of zinc by the intestines is important, especially for infants, to warrant the mechanical barrier function of this layer and, with this, protection from invading pathogens and prevention of other mucosa-related diseases [36]. Moreover, in the airway and intestinal mucosa of humans, zinc also modulates cytokine production and cell-mediated immunity and acts as a potent antioxidant and anti-inflammatory modulator [3, 44]. The involvement of zinc in the abovementioned processes in human mucosa match with the overlapping processes we identified in the intestinal mucosa of piglets and in IPECJ2 enterocytes. Therefore, our in vivo data and set of ‘putative’ effector and regulatory genes, among which hypoxia-modulating genes like HMOX1^*E*^ and MTs^*E*^, several cytokines and genes that may regulate cytokine expression (e.g. MT1A^*E*^ and ZFP36L2), may provide further insight about the complex biological mechanisms underlying the prophylactic and therapeutic effect of zinc in humans.

### Rye

In response to rye, cell cycle/mitosis and glycan synthesis were stimulated both in the jejunum of broilers as well as in IPEC-J2 cells. This is in line with our observation that villus length and crypt depth at 21 days were increased in broilers fed with the 10% rye diet [57]. Enhanced mitosis and synthesis of glycans, the major component in the mucus layer, may support renewal of epithelial cells and, with this, results in increment of the net surface that absorbs nutrients. This surface increment may compensate for the inefficient uptake of nutrients from the viscous digesta imposed by a rye-rich diet. Despite this, we observed regulation of processes related to glycan synthesis and expression of mucins, whereas histological analysis showed no increase in mucus-producing goblet cells in the jejunum of our broilers, as was observed in a recent study in broilers fed with rye-rich diet [54]. In the intestinal tissue of mice, mTor signalling controlled differentiation of goblet and paneth cells [66]. However, we were not able to identify DEGs coding for ‘secreted’ effector proteins (only non-secreted, e.g. PTEN^*E*^; see the ‘Results’ section) within our IPEC-J2 datasets of genes that have potential to steer this process. With respect to the abovementioned mucus production, it was reported that PTEN-AKT/mTOR signalling modulates IL9-mediated mucus production in lung epithelial cells [24, 38]. In our in vivo study, at day 21, the ‘IL9 signalling pathway’ was called significant in the broilers fed with the 10% diet [57]; however, we observed no higher gene expression of IL9 itself in vivo, nor in IPEC-J2 cells.

Ovarian granulose cells form a protective mucus layer around the oocyte and produce steroid hormones (including sex hormones) and INHA protein by a similar BMP/SMAD transcriptional mechanism that activates calcium deposition by osteoblast [1]. Activation of activin receptors by inhibins stimulated differentiation of gastric mucosal cells with accumulation of mucous granules (4a). Based on this profile of TGF-specific effectors, we propose that transcription mediated by BMP, TGF-beta and activin receptors could affect the following processes in the intestinal layer and perhaps also (indirectly) in the periphery: (i) abrogation of cell cycle arrest via regulation of the TNF-SMAD-ZFYVE16-MYC axis resulting in relief of repression by CDKNs; (ii) (de)regulation of Ca^2+^ deposition in a similar fashion as by osteoblasts; and (iii) INHA-regulated cell differentiation involved in formation of a mucus layer by a mechanism related to that observed for developing oocytes in association with ovarian granulose cells.

In a recent in vivo study, it was reported that a high dietary inclusion of rye (580 g/kg) reduced bone strength and bone mineralisation/calcium deposition in broilers [55]. Demineralisation of bones (osteopenia and osteoporosis) is frequently observed in humans with ‘gluten-sensitive enteropathy’ (coeliac disease) [64]. Therefore, it could be interesting to investigate whether a diet with inclusion of rye, containing a high concentration of gluten, or other related plant allergens, also influence the calcium concentration in the shell of eggs [33]. If so, measurement of the calcium levels in egg shells may be a relatively straight forward and animal-friendly manner (model) to study the effect of human diets containing gluten or other coeliac-inducing plant allergens on bone demineralisation [15].

Additional research is needed to obtain more detailed information about the role of the here predicted set of effectors secreted by enterocytes (i.e. antagonists and activators of BMP, TGF-beta and activin receptors) in calcium homeostasis, and of MUC4^*E*^, MUC13^*E*^ and B3GALT1^*E*^ in mucus formation. Because several of the above described effectors can inhibit, as well as stimulate TGF-beta-mediated transcription, no firm conclusions can be drawn about activation or inhibition of cell division in vivo. Only the observed increased villus length and crypt depth indicated that stimulation of renewal of epithelial cells in the intestine layer of broilers had occurred before day 21, perhaps to repair mucosal damage induced by shear forces imposed by the high viscosity of a rye-rich luminal content [54].

### Amoxicillin

For amoxicillin, the overlap in biological processes between broilers and IPEC-J2 cells was restricted to the GO-term ‘microtubule cytoskeleton’. Bioinformatics analyses indicated that several genes that responded in vivo at both test days encode proteins involved in depolymerisation of MTub filaments. Several of these proteins also interacted with UBC, suggesting that proteasomal degradation of these MTub-associated proteins contributes to execution of the depolymerisation process. In response to cell stress, depolymerisation and degradation of MTubs is part of a recycling mechanism of cells. STMN1 is a key regulator of MTub depolymerisation. STMN1 destabilises microtubules, prevents their assembly and promotes disassembly of microtubules. In hypoxic (stressed) cells, phosphorylation of STMN1 by MAPK14 inactivates these functions and prevents depolymerisation of MTub filaments [29]. Also, several studies in cancer cells showed that microtubule remodelling occurred under hypoxic conditions, and that HIF1A transcriptional activity mediated beta-tubulin expression [46]. Alteration in microtubule network/structures affects the transport of HIF1A to the nucleus, its proteasomal degradation and localization in organelles (reviewed in [42]), and transcriptional activity of HIF1A is dependent of intact microtubules [2]. Gene expression of OSER1, a biomarker for hypoxia, was downregulated in the intestine of broilers at day 5 after hatch. This lower expression may be a reaction on an earlier elevated expression level, suggesting that amoxicillin administration during the first 24 h after hatch may have induced oxidative stress, as it did in IPEC-J2 cells within 2 h and in vivo when administrated prophylactically to female dogs after surgery [47]. The observed gene expression pattern of the HIF1A-transcribed vasodilators HMOX1^*E*^ and NPPA^*E*^, vasoconstrictors EDN1^*E*^ and the sugar-metabolising enzymes HK2^E^ and PDK1^*E*^ [17, 27] induced in IPEC-J2 cells by amoxicillin indicated that enterocytes may play a pivotal role in restoring normoxia in the intestine. Therefore, MTub (de)polymerisation and HIF1A-mediated transcription of effector genes by enterocytes to rescue cells in the intestine from oxidative stress may be tightly linked processes that could have occurred in the intestine of broilers in the first 24 h after hatch.

Interestingly, it was shown that gene expression of STMN1 was correlated with genetic variation in the loci coding for EGFR receptors [58]. EGFR ligand EREG also steers Aurora kinase-mediated signalling, resulting in stimulation of mitotic spindle assembly in a microtubule-dependent manner [23, 56]. Promotion of cell division in response to amoxicillin-induced cell death (apoptosis) may be needed to repair a damaged epithelial layer. Perhaps cell division to repair the epithelial layer is regulated in a MYC-dependent manner (see also above) with a pivotal role for the in vivo downregulated overlapping DEG ZBTB17. Less binding of ZBTB17 to MYC results in less relief of transcriptional repression of cyclin-dependent kinase inhibitors and may stimulate cell division and/or prevent apoptosis. Noteworthy, lactams and analogues of these natural compounds are potent anticancer drugs that destabilise tubulins, most of them interfering with the formation of the mitotic spindle, resulting in death of tumour cells [20, 34, 40]. A disadvantage of these drugs is their toxicity for non-tumour cells. Because most beta-lactams, including amoxicillin, are non-toxic, high doses can be administrated without provoking adverse effects. The potential of this group of antibiotics as anticancer drugs in humans is recognised in the USA, and several projects of the National Institute of Health were launched recently to investigate this (http://projectreporter.nih.gov/project_info_description.cfm?aid=7912932; click on the ‘similar tab’ for related projects). The information about the effector genes and cognate overlapping processes induced by amoxicillin in this study may be useful to select markers and/or targets to evaluate potential of beta-lactams as anticancer drugs.

With regard to the gut and digestive physiology, chickens differ more from humans than pigs do. For chickens, an enterocyte cell line is described [11]. This cell line is not clonal, difficult to grow/maintain, and monolayers are vulnerable to subtitle changes in the culture medium, making these cells unsuitable for nutritional intervention studies. The results of our study showed that integration of data obtained with the porcine IPEC-J2 cells also provided additional insight in biological processes/pathways activated in the intestine of chickens. Therefore, our approach of pre-screening in IPEC-J2 cells combined with an in vivo trial in chickens may be a cheap alternative for expensive (dietary) intervention trials in pigs, also for testing of specific groups of (dietary) interventions (e.g. plant allergens and beta-lactams; see above) important for human health.

## Conclusions

In the present study, we showed that the set of effector ‘early’ signalling molecules/proteins expressed by IPEC-J2 cells in response to zinc, rye and amoxicillin could be relevant for steering these biological processes in the intestinal mucosa of monogastric vertebrates, including humans. We conclude that pre-screening of dietary interventions in this IPEC-J2 bioassay may provide additional insight in intestinal gene regulation mechanisms imposed by functional (non-strict nutritional) human and animal dietary interventions. This insight creates opportunities to conduct in vivo intervention trials in a more focused and effective manner, with fewer test animals, and translatable to human intestinal homeostasis.

## Additional files

Additional file 1: Materials and methods supplement. (DOCX 15 kb)

Additional file 2: Table S2. The expression data, and derived list of DEGs, used for functional analysis of the ZnO, rye and amoxicillin in vivo, and IPEC-J2 in vitro interventions. (XLSX 292 kb)

Additional file 3: Table S3. Full names of overlapping DEGs expressed in vivo and in vitro ZnO, rye and amoxicillin interventions. (XLSX 12 kb)

Additional file 4: Table S4a In vitro and in vivo DEGs associated with PAK-mediated steering of cell adhesion, adherence and tight junction processes. Table S4b Pathway mapping of in vitro and in vivo DEGs associated mediated PAK-mediated steering of cell adhesion/adherence and tight junction processes. (XLSX 18 kb)

## Abbreviations

CC_FAT: Cellular component gene ontology term
CSF: Colony stimulating factor
DEGs: Differentially expressed genes
DMEM: Dulbecco’s modified Eagle’s Medium
FC: Fold change
FCS: Foetal calf serum
GEO: Gene expression omnibus
GO-term: Gene ontology term
HAMs F10: Nutrient mixture F-10
HUGO: Gene Nomenclature and symbols of human gene names in the HGNC database. For abbreviations of genes (HUGO official gene symbols), we refer to the ‘GeneCards’ (Weizmann Institute of Science) and the NCBI Gene reports (Entrez) databases.
IL: Interleukin
M24: 24-well tissue culture plate
MTub: Microtubule filaments
TNF: Tumour necrosis factor
