# Supplementary material for: Enrichment of in vivo transcription data from dietary intervention studies with in vitro data provides improved insight into gene regulation mechanisms in the intestinal mucosa
Source: Genes Nutr. 2017 Apr 13;12:11. doi: 10.1186/s12263-017-0559-1 (PMC5390468; doi:10.1186/s12263-017-0559-1)
Supplement: Supplementary file 1 — Materials and methods supplement. (DOCX 15 kb) [file 12263_2017_559_MOESM1_ESM.docx]

**Additional file 1**; Materials and Methods

**A) Brief description of the three intervention studies used for comparison with IPEC-J2 data sets.**

Details about the composition of the diets supplemented with additives, the control diets, sampling of biological material for analysis, isolation of total RNA from these tissues, and microarray analysis and extraction of differential expressed genes (DEGs) were described in reports available on-line or in the articles published about these trails [Schokker et. al 2014a, Krimpen et. al 2015, Jansman et. al 2016 and Schokker et. al 2017 submitted].

-Zinc study in pigs: Weaned pigs were first fed a regular diet containing 60-100 mg/kg zinc oxide from day 0 to 14 after weaning. Secondly, a higher dose of zinc oxide (2500 mg/kg) was fed from day 14 to 23 post weaning. Lastly, all pigs were fed the regular amount of zinc oxide again from day 23 to 35. The pigs were housed in three pens with 12 pigs per pen per treatment. At day 14, 23 and 35, 6 pigs from each pen were euthanized for biological samples, including mucosal scrapings from jejunal and ileal tissue. RNA pools (n=6) of jejunal and ileal tissues were subsequently analysed by (single colour) Agilent porcine microarrays. The gene expression dataset was statistically analysed by the R package LIMMA [Schokker et.al. 2014b] and the produced output files were up- and down-regulated gene lists for the comparisons of interest.

-Rye study in broilers: In this animal experiment 960 one-day-old male broiler chickens were housed in 24 pens, each pen contained 40 chickens. The broiler chickens were fed different inclusion levels of rye of 0%, 5% and 10% weight/volume in the feed. On day 14, 21 and 28, 6 chickens from each pen were dissected for biological sampling. RNA pools (n=6) of jejunal tissue was analysed by (single colour) Agilent Gallus Gallus microarrays. In a similar manner as described above, lists of up- and down-regulated genes were generated.

-Antibiotic study in broilers: The antibiotic was administrated on day 1 after hatch for 24 hours via the drinking water (67 mg Octacillin/L water). At day 1 (controls only), day 5 and day 14 of age, per time-point 80 chickens were dissected. Measurements were performed for either control or antibiotic treated broiler chickens and jejunal tissue was analysed. In a similar manner as described above, lists of up- and down-regulated genes were generated.

**B) Labelling, hybridization, scanning and feature extraction of microarrays.**

Briefly, 500 ng RNA of each sample was labelled with the One-Color Microarray-Based Gene Expression Analysis Low input Quick Amp Labelling kit and 600 ng of Cy3 labelled cRNA was used for hybridisation on each patch. Hybridisation and washing of the arrays was performed according to the protocol provided by Agilent Technologies for the One-Color Microarray-Based Gene Expression Analysis Low input Quick Amp Labelling kit. Duplicate RNA samples were labelled and hybridized separately to obtain 2 biological replicates. Arrays were scanned using a DNA microarray scanner with Surescan high resolution Technology (Agilent Technologies). Agilent Scan Control with resolution of 5 µ, 16 bits and PMT of 100%. Feature extraction was performed using protocol 10.7.3.1 (v10.7) for 1 colour gene expression. The files generated by the feature extraction software were loaded in GeneSpring GX 9.0.5, in which a log2-transformation and a median normalization (75 percentile) was performed on all probes. Probes with a raw intensity of <60 (flooring) and with a corrected p-value of >0.05 (OnewayANOVA significance analysis with asymptotic p-value computation) were filtered out of the data files.
